# Case of Dysphonia

**Published:** 1844-07

**Authors:** 


*Case of Dysphonia.*—The sister of J. R., Esq., of this city, an estimable lady, who for several weeks had been unable to speak, except in a faint whisper, having tried some remedies in vain, consulted me. I directed her to rub three drops of Croton oil along the trachea; and having done this, she had, in a few hours, sense of constriction about the upper part of

---

the thorax, followed by "two" expectorations of sanguineous mucus. Shortly after this, her voice became quite natural. This case is the only one in which I have ever observed the least unpleasant effect.—*N. Y. Jour. Med.*, for May.

